# Etiologies and Outcomes of Diabetic Ketoacidosis in Cancer Patients: A Retrospective Analysis

**DOI:** 10.3390/cancers17172728

**Published:** 2025-08-22

**Authors:** Ayush Gandhi, Rebecca Jeun, Zhongya Wang, Sonya Khan, Conor Best, Victor Lavis, Sonali Thosani

**Affiliations:** 1 Department of Hospital Medicine, The University of Texas MD Anderson Cancer Center, Houston, TX 77030, USA; agandhi2@mdanderson.org; 2 Department of Endocrinology, Diabetes and Nutrition Care, University of Louisville Health, Louisville, KY 40202, USA; rebecca.jeun@louisville.edu; 3 Department of Biostatistics, The University of Texas MD Anderson Cancer Center, Houston, TX 77030, USA; zwang28@mdanderson.org; 4 Department of Endocrine Neoplasia & Hormonal Disorders, The University of Texas MD Anderson Cancer Center, Houston, TX 77030, USA; sskhan1@mdanderson.org (S.K.); cjbest@mdanderson.org (C.B.); vrlavis@mdanderson.org (V.L.)

**Keywords:** diabetic ketoacidosis (DKA), cancer related hyperglycemia, euglycemic DKA, steroid induced hyperglycemia, oncology endocrinology, cancer treatment complication, glucose monitoring in cancer

## Abstract

Background/Objectives: Diabetic ketoacidosis (DKA) is a life-threatening emergency resulting in significant morbidity and health care utilization. The most common cause of DKA in the general population is insulin nonadherence, but limited data exists on precipitating factors for DKA in cancer patients. In this study, we characterize the demographic and clinical characteristics of hospitalized patients with DKA at our comprehensive cancer center. Methods: This single-center retrospective study evaluated 91 patients with 94 admissions for DKA at our institution between January 2019 and December 2021. Demographic, clinical, and biochemical data were obtained from a review of the electronic medical record. Patient characteristics were summarized using descriptive statistics for continuous variables and categorical variables. Results: Of the 91 patients, 21% of patients (*n* = 19) had underlying type 1 diabetes, 49% of patients (*n* = 45) had type 2 diabetes, and 30% of patients (*n* = 27) had drug-induced diabetes. A total of 39% (*n* = 29) had poorly controlled diabetes with HbA1c > 9% (75 mmol/mol). In patients with known type 1 diabetes, the most common provoking factors were inadequate insulin therapy and infection related causes. In patients with known type 2 diabetes or no previous history of diabetes, the most common provoking factors were medications such as immune checkpoint inhibitors, SGLT2 inhibitors and steroids. Conclusions: We characterized the precipitating factors for DKA based on patients’ underlying diabetes status. While insulin non-adherence was the most common cause in patients with known type 1 diabetes, drugs associated with cancer related treatments emerged most common precipitating factor for DKA in cancer patients with underlying type 2 diabetes or drug induced diabetes. A tailored approach with proactive counseling may be helpful in timely recognition and treatment of DKA in cancer patients.

## Simple Summary

Diabetic ketoacidosis (DKA) is a serious and often under-recognized complication in cancer patients. Unlike the general population, cancer patients may develop DKA due to treatment related factors, including high dose steroids, immune checkpoint inhibitors and Sodium Glucose Co-Transporter 2 (SGLT) inhibitors. In this retrospective study, we characterized the precipitating causes of DKA in a cohort of hospitalized cancer patients, many of whom had no prior history of diabetes. Euglycemic DKA was also commonly observed in patients receiving SGLT2 inhibitors, particularly in the context of nausea, vomiting or poor oral intake. Oncologists should remain vigilant for new or worsening hyperglycemia in patients receiving therapies known to induce DKA. Early recognition and proactive counseling may help prevent life threatening emergencies.

## 1. Introduction

Patients with cancer have a high prevalence of diabetes, with approximately 20% of cancer patients living with diabetes [1]. Published studies have confirmed that poorly controlled diabetes can adversely impact cancer treatment and outcomes leading to higher infection rates and mortality [1,2,3]. Diabetic ketoacidosis (DKA) is a life-threatening complication of diabetes characterized by hyperglycemia, ketosis, and metabolic acidosis, with risk for substantial morbidity with delayed diagnosis and treatment. In the general population of patients with diabetes, the most common precipitating factors for DKA are inadequate insulin therapy (often due to nonadherence) and infection [4,5,6,7].

In cancer patients, there are other potential contributors to the development of hyperglycemic emergencies. These include exposure to drugs known to cause hyperglycemia through a variety of mechanisms. These drugs include glucocorticoids, mTOR (mammalian target of rapamycin) inhibitors, AKT (Protein Kinase B) inhibitors, PI3K (Phosphoinositide 3-kinase) inhibitors, tyrosine kinase inhibitors, MEK (mitogen-activated protein kinase) inhibitors, peg-asparaginase, BCL-2 (B-cell lymphoma 2) inhibitors, brentuximab and androgen deprivation therapy [8,9,10,11]. Immune checkpoint inhibitors cause a unique form of fulminant insulin-deficient diabetes through immune mediated beta cell destruction. Diagnosing DKA in cancer patients can be challenging, as symptoms like nausea, vomiting, and malaise may be misattributed to chemotherapy side effects or the cancer itself. Cancer patients who develop DKA are often at higher risk of severe dehydration due to poor nutritional intake or gastrointestinal losses from treatment toxicity or infection related to immunosuppression from chemotherapy or the underlying malignancy. These factors may compound the severity of DKA.

Given the paucity of literature on DKA in adult cancer patients, we performed a retrospective analysis to characterize the precipitating factors, clinical course, and outcomes of DKA in this unique population. By identifying the etiologies and outcomes of DKA in cancer patients, we aimed to inform targeted strategies to prevent and manage DKA in oncology settings [4,5,6,7].

## 2. Materials and Methods

### 2.1. Settings and Participants

This was a single-center, retrospective study of hospitalized patients with DKA between January 2019 and December 2021 at the University of Texas MD Anderson Cancer Center, one of the largest comprehensive cancer centers in the United States. We included patients with cancer who had admission or discharge diagnoses corresponding to the International Classification of Diseases, Tenth Revision (ICD-10) codes for Type 2 diabetes mellitus with ketoacidosis (Ell.1x), Type 1 diabetes mellitus with ketoacidosis (E10.1x), or Other specified diabetes mellitus with ketoacidosis (E13.1x). The diagnosis of Type 1 diabetes was further confirmed by an endocrinologist based on clinical presentation/history, the presence of auto-antibodies, or low C-peptide levels.

### 2.2. Data Collection

We collected data from electronic medical records on age, sex, cancer type and stage, diabetes type (pre-existing type 1 or type 2, or drug induced diabetes), most recent HbA1c prior to admission, and home diabetes regimen. We looked at HbA1c results that were recorded during the admission and within 3 months prior. If a patient had an HbA1c done in both instances, we utilized the most recent value closest to the DKA admission. We recorded precipitating factors for DKA as documented in notes and by laboratory/imaging findings. Medication triggers were defined as usage of agents known to affect glucose metabolism in the period leading up to DKA, such as high-dose steroids, immunotherapy, targeted therapies, etc. Inadequate insulin therapy category was broadly defined to include instances related to patient noncompliance for variety of reasons, reduction of insulin dosage due to poor oral intake and iatrogenic causes [12].

Patients were categorized as having a “lack of diabetes survival skills” if they did not make adequate adjustments to their insulin dosing to prevent DKA or if they inappropriately held their insulin, as documented by the Endocrinology team upon initial evaluation of the DKA event.

### 2.3. Definitions

DKA was defined according to the American Diabetes Association (ADA) consensus criteria: blood glucose > 250 mg/dL (13.89 mmol/L) except in cases of SGLT2 inhibitor-associated euglycemic DKA where no minimum glucose was required, anion gap metabolic acidosis (serum bicarbonate < 18 mEq/L (18 mmol/L) and/or arterial pH < 7.30 with an anion gap > 10 mEq/L (10 mmol/L), and the presence of ketonemia (serum β-hydroxybutyrate > 3 mmol/L) or moderate-to-large ketonuria [12]. DKA severity was stratified as mild (bicarbonate 15–18 mmol/L, pH 7.25–7.30), moderate (bicarbonate 10 to <15 mmol/L, pH 7.00 ≤ 7.24), or severe (bicarbonate < 10 mmol/L, pH < 7.00), in accordance with ADA guidelines. We defined “poor baseline glycemic control” as HbA1c > 9% (75 mmol/mol) prior to admission.

“Drug-induced diabetes” was defined as diabetes that developed in patients with no prior history of the condition, with onset directly attributed to a therapeutic agent. We reviewed patients notes at the time of diagnosis to verify if they had previously undiagnosed diabetes and categorized them accordingly. Patients with underlying type 1 and type 2 diabetes were classified as such, and those without a history of diabetes who developed DKA were categorized as having drug induced diabetes.

“Steroid induced hyperglycemia” was identified in patient with documented exposure to steroids, which were considered a contributing cause to their hyperglycemia. We also looked for common chemotherapeutic agents known to cause hyperglycemia, such as PI3 kinase inhibitors, enfortumab, etc. If patients were not on these agents, they were not categorized having chemotherapy-associated hyperglycemia.

### 2.4. Management

All patients were treated with intravenous fluids, insulin infusion, and electrolyte repletion per ADA recommendations [12]. Transition to subcutaneous insulin was done once ketoacidosis had resolved and oral intake was resumed.

### 2.5. Statistical Analysis

Patient characteristics were summarized using standard descriptive statistics, including means, standard deviations, medians, quartiles, and ranges for continuous variables and percentages for categorical variables. Comparison of patient characteristics was conducted by underlying diabetes type. For the continuous variables, one way ANOVA or Kruskal–Wallis test was used while Fisher’s exact test was used to compare categorical variables. The presence of a significant difference among groups was indicated by a *p*-value less than 0.05.

To identify independent predictors of in-hospital mortality, univariate survival analysis was performed, with time to event defined as the number of weeks from admission to in-hospital death. Survival probabilities were estimated using the Kaplan–Meier method, and differences in survival curves across covariates of interest were evaluated using log-rank test. Associations between survival outcomes and clinical variables were further examined using Cox proportional hazard regression models. Covariates of interest included age at admission, length of hospital stay, cancer stage (Metastatic vs. Non-Metastatic), type of diabetes mellitus, and classification of provoking factors.

All analyses were conducted using SAS version 9.4 and SAS Enterprise version 7.15 HF7. No adjustments for multiple testing were included. The study was reviewed and approved by the institutional review board with a waiver of informed consent due to its retrospective design.

### 2.6. Confounding Factors

To account for potential confounding factors, we measured several key variables. We used the index DKA admission during the study period as our main anchoring point, and looked at primary site and stage of cancer closest to DKA admission. Infections were identified based on documented infectious diagnoses in clinical notes or the hospital problem list and were considered active if they occurred within 48 h of admission. All variables were abstracted from the electronic medical record, with a review of clinical notes, laboratory results and culture data to ensure accuracy.

## 3. Results

### 3.1. Baseline Characteristics

Ninety-one cancer patients accounted for ninety-four episodes of DKA over the three-year period. Key baseline characteristics are shown in Table 1. The median age at DKA presentation was 63 years (IQR 44–69), and 46% of the patients (*n* = 42) were women. 21% of the patients (*n* = 19) had type 1 diabetes mellitus, 49% (*n* = 45) had type 2 diabetes, and 30% (*n* = 27) had drug-induced diabetes. Among 74 patients with HbA1c measured prior to admission, the median HbA1c was 8.2% (66 mmol/mol) (IQR 7.3–10.4% (56–90 mmol/mol)), and 39% of the patients (*n* = 29) had poor baseline glycemic control (HbA1c > 9% (75 mmol/mol)) (Table 1).

The most common types of malignancies in the cohort were gastrointestinal cancers (20%), dermatologic cancers (15%), followed by hematologic malignancies (14%), and genitourinary cancers (11%). Lung cancers and breast cancers each accounted for 8% of cases. Head and neck cancers and gynecologic cancers were less common (5% each).

A total of 67% of the entire cohort (*n* = 61) had Stage IV cancer at the time of the DKA event, comprising. A total of 93% of the patients (*n* = 25) with drug induced diabetes, 58% of the patients (*n* = 11) with type 1 diabetes and 56% of the patients (*n* = 25) with type 2 diabetes (Table 2).

**Table 3:** Antihyperglycemic Therapies.

Medication Regimen on Admission	Total (*n* = 91)	Type 1 Diabetes (*n* = 19)	Type 2 Diabetes (*n* = 45)	Drug Induced Diabetes (*n* = 27)
None—no. (%)	30 (33%)	0 (0%)	6 (13%)	25 (93%)
Oral/GLP1agonist—no. (%)	17 (19%)	0 (0%)	17 (38%)	0 (0%)
Oral/GLP1 agonist + basal insulin—no. (%)	9 (10%)	0 (0%)	9 (20%)	0 (0%)
Oral/GLP1 agonist + multiple dose insulin—no. (%)	3 (3%)	1 (5%)	1 (2%)	0 (0%)
Multiple dose insulin—no. (%)	24 (27%)	13 (68%)	10 (22%)	1 (4%)
Insulin pump—no. (%)	8 (9%)	5 (26%)	2 (4%)	1 (4%)

Only 4 patients were using continuous glucose monitoring (CGM) prior to presentation: three with type 1 diabetes and one with type 2 diabetes.

We also evaluated the use of anti-depressants in our patient population as diagnosis of depression is associated with higher A1c levels and patients with depression are more likely to skip insulin, which could increase risk of DKA [13,14]. Antidepressant use was relatively common, 23% of patients (*n* = 21). The distribution of antidepressant use was similar across different cohorts, 21% of patients (*n* = 4) in type I diabetes group, 20% of patients (*n* = 9) in type II diabetes group and 30% of patients (*n* = 8) in Drug induced diabetes group (Table 4).

### 3.2. Episodes of DKA

DKA severity was mild in 41% of episodes (*n* = 39), moderate in 28% of episodes (*n* = 26), and severe in 31% of episodes (*n* = 29). While mild DKA was the most common overall presentation in patients with known type 1 or type 2 diabetes, more than 30% of patients in each subgroup presented with severe DKA. There was no difference in DKA severity among the types of diabetes. The median length of hospitalization varied significantly by diabetes status (*p* = 0.019), with patients with type 2 diabetes experiencing the longest median stay (8 days; IQR: 4–16), followed by patients with drug induced diabetes (6 days; IQR: 4–10), and those with type 1 diabetes (4 days; IQR: 3–6) (Table 5)

The all-cause mortality rate during the index DKA hospitalization was 16% of patients (*n* = 15). While DKA was not identified as the cause of death, patients died during hospitalization after DKA resolution due to advanced cancers or associated complications such as sepsis or multi-organ failure. Death during the DKA hospitalization occurred in 24% of the patients (*n* = 11) with type 2 diabetes, 14% of the patients (*n* = 4) with drug induced diabetes, and none of the patients with type 1 diabetes (*p* = 0.055). By the end of the study period, 44% of patients (*n* = 40) had died due to progressive cancer or related complications. Patients with advanced malignancies had a markedly worse prognosis (Table 6).

### 3.3. Predictors of In-Hospital Mortality

To identify factors associated with in-hospital mortality, we performed a univariate survival analysis on 40 patients who died during the study period, 15 of whom died during the index hospitalization. As shown in Table 7, length of hospital stay and age were not significantly associated with mortality (HR = 1.00, 95% CI: 0.98–1.02, *p* = 0.79; HR = 1.02, 95% CI: 0.98–1.05, *p* = 0.36, respectively). While cancer staging did not show significant differences across detailed categories (*p* = 0.39), patients with metastatic disease showed as a trend toward higher mortality compared with non-metastatic disease (HR = 4.4, 95% CI: 0.6–33.9, *p* = 0.10).

**Figure 1:**
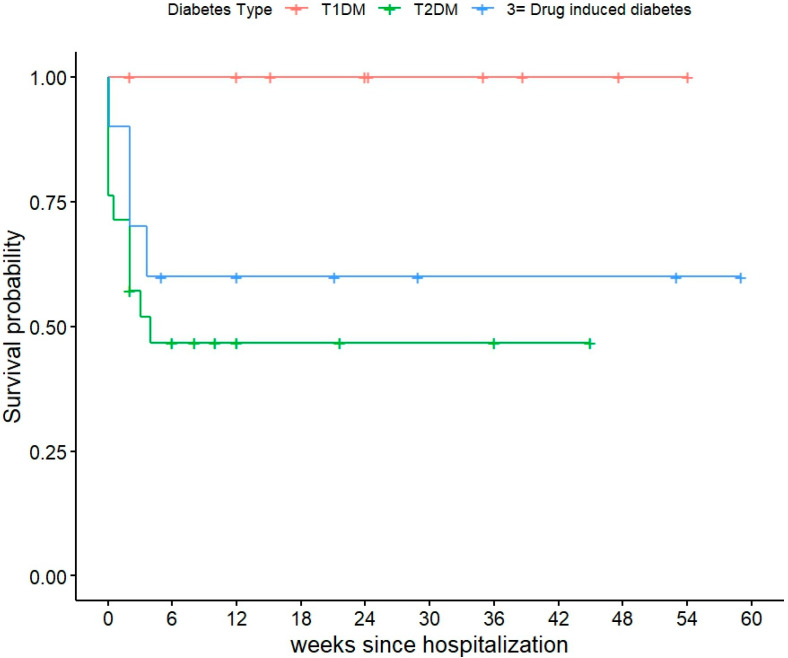
Survival curves among the diabetes type.

A significant difference in survival was also observed by diabetes type (*p* = 0.035) (Figure 1). Notably, there were no in-hospital deaths among patients with type 1 diabetes, whereas patients with type 2 diabetes and drug induced diabetes experienced substantial mortality. Due to absence of events in the type 1 diabetes group, the hazard ratios for this variable were not usable. Provoking factors, including drug-induced or infection-related causes, were not significantly associated with survival. We were unable to perform a multivariate analysis of contributing factors to mortality given the low number of events.

### 3.4. 30-Days Post Hospital Mortality

The univariate survival analysis for 30-day mortality after discharge (25 patients, 5 events) did not reveal any statistically significant associations. Median survival was not estimable for most groups due to limited events. Neither length of hospital stay (HR = 1.00, *p* = 0.90) nor age (HR = 1.01, *p* = 0.75) showed significant effects. Among diabetes categories, T2DM (HR = 2.82, *p* = 0.81) and no diabetes (HR = 1.50, *p* = 0.08) did not differ significantly from T1DM (Table 8)

### 3.5. Provoking Factors for DKA

A total of 84% of DKA episodes (*n* = 79) had a single clear precipitating factor identified, whereas 16% patients (*n* = 15) had DKA attributable to a combination of factors. Table 9 summarizes the precipitating causes of DKA in our cohort. In the entire cohort, drug related causes were the primary provoking factor in 53% of DKA episodes, making this the most common precipitating factor in our patients. Among drugs contributing to DKA, immune checkpoint inhibitor (ICI) therapy was implicated in 29% (*n* = 26) of all episodes (Table 4). High-dose corticosteroid therapy was another frequent contributor at 11%, followed by less common drugs such as PI3K inhibitors and peg-asparaginase. Inadequate insulin therapy was the second most common precipitating factor, accounting for 36% (*n* = 34) of DKA episodes. This category included patients who missed insulin doses or did not appropriately intensify insulin despite rising glucose, as well as a subset of cases where basal insulin therapy was inappropriately held upon hospital admission. Infection was a precipitant in 21% (*n* = 20) of DKA cases.

Analyzing precipitating causes by diabetes subgroups provided further insights. For patients with type 1 diabetes, the predominant precipitant was insulin omission or under-dosing. A total of 67% of patients (*n* = 12) with type 1 diabetes did not adjust their basal insulin when they became unable to eat due to chemotherapy side effects, deliberately skipped doses because of misunderstanding how to manage insulin during illness or failed to seek medical advice as their glucose control worsened prior to hospitalization. Infection was the next most common trigger in patients with type 1 diabetes, contributing to DKA in about 20% of those patients (*n* = 4).

For patients with type 2 diabetes, medication-related precipitating factors dominated. 27% of DKA episodes (*n* = 12) were related to SGLT2 inhibitors, 16% patients (*n* = 7) had DKA episodes related to steroid use, and 7% patients (*n* = 3) had DKA episodes related to immunotherapy use. (Table 4) Poor oral intake from illness or chemotherapy combined with continued SGLT2 inhibitor likely precipitated ketosis in patients with type 2 diabetes. Infections and failure to adjust insulin therapy to rising glucose levels were also significant precipitating factors in patients with type 2 diabetes.

For patients without a previous history of diabetes, cancer treatment-related factors were the primary triggers for DKA. In this group, immune checkpoint inhibitor (ICI) therapy was the single most frequent precipitant, in line with the high fraction of ICI-associated diabetes cases noted earlier. Other oncologic treatments causing DKA in patients without prior diabetes included PI3K inhibitors and peg-asparaginase, which is known to induce pancreatitis and hyperglycemia. Many of these patients had significant tumor burden and had received multiple lines of therapy. There were 7% patients (*n* = 2) for whom a combination of concurrent infection and cancer related drug use was the precipitating factor for DKA.

## 4. Discussion

DKA is a severe and costly acute complication of diabetes. In the general population, DKA hospitalization rates have been rising over the past two decades, even as overall in-hospital mortality for DKA has declined from around 1.1% in 2000 to 0.4% in 2014 [15]. While there are several case reports and one small case series published on DKA in cancer patients [16], to our knowledge this report represents the largest case series identifying the precipitating factors and outcomes of DKA in cancer patients to date.

In a real-world cohort study examining the prevalence of prediabetes and diabetes in patients with cancer, gastrointestinal malignancies were the most common among patients with known diabetes, followed by genitourinary, respiratory and breast cancer [17]. Similarly, in our cohort, the most common malignancies were also gastrointestinal followed by dermatological, hematological, and genitourinary malignancies. It is possible that results were confounded by the fact that patients with gastrointestinal malignancies were more likely to receive therapies that included SGLT2 inhibitors and/or immune checkpoint inhibitor (ICI) therapy, which were found to be major contributor to DKA development in our study. Similarly, patients with dermatological malignancies had higher use of ICI therapy, which leads to an increased risk of DKA. Patients with hematological malignancies may have increased DKA risk due to chemotherapy regimen that include high dose steroids, which are often used as premedications and likely increase risk of hyperglycemia and DKA. Inadequate insulin therapy was a precipitating factor for 36% of DKA episodes (*n* = 34). While most of these episodes were due to omission of insulin by patients, 5 were attributable to failure of hospital staff to provide treatment with basal insulin on admission to the hospital or after discontinuation of insulin pump and therefore were preventable. Tools within the electronic medical record for identification of patients with absolute insulin deficiency and specialized insulin order forms may reduce the likelihood of these events, particularly in a center such as ours, where the medical and nursing staff are focused on the complexities of treatment of cancer [18].

One key finding from our study is that the profile of precipitating causes in cancer patients differs markedly from the general diabetic population. ICIs were a particularly prominent precipitant, accounting for 29% of all DKA episodes. ICI-induced diabetes is an immunologically mediated form of diabetes characterized by absolute insulin deficiency, frequently fulminant in onset and presenting with DKA. In our cohort, ICI therapy precipitated DKA both in patients with and without pre-existing diabetes [19,20]. In a prior analysis of ICI-DM cases at our cancer center [21], 69% of those patients presented with DKA or hyperosmolar hyperglycemic syndrome, which is consistent with reports from other institutions [22]. Our findings reinforce that oncologists and endocrinologists should maintain a high index of suspicion for new onset hyperglycemia with polyuria, polydipsia and/or weight loss in patients on ICIs, even those without a diabetes history, as early recognition of ICI-DM can be lifesaving.

Infection and sepsis were precipitating factors in 20% of our cases of DKA. Infections can induce DKA by increasing counter-regulatory stress hormones and causing insulin resistance, and cancer patients are particularly susceptible given their immunosuppressed status [1].

Use of SGLT2 inhibitors precipitated DKA in 13% (*n* = 12) of the overall cohort and contributed to 27% of DKA episodes (*n* = 12) with type 2 diabetes. SGLT2 inhibitors promote glycosuria and a shift toward ketone metabolism, and they have been associated with euglycemic DKA in patients with type 1 and type 2 diabetes [23,24,25]. While these drugs are known for their cardioprotective and renal benefits, they must be used with caution in patients with cancer. Patients being treated for cancer may have poor oral intake, nausea, or vomiting due to chemotherapy, leading to volume depletion and carbohydrate restriction, which in turn predisposes to euglycemic ketoacidosis. In our cohort, patients with type 2 diabetes on SGLT2 inhibitor therapy developed DKA during such vulnerable periods. Cancer patients should be counseled to hold their SGLT2 inhibitors when they are ill or cannot eat or drink normal amounts of fluids. Ensuring adequate hydration and monitoring for ketosis in patients who continue SGLT2 inhibitors is critical. We recommend extensive counseling and careful monitoring of any cancer patient on an SGLT2 inhibitor, with guidance on sick-day management including recognition of early signs of ketosis or volume depletion and discontinuation of the medication when appropriate.

Glucocorticoids contributed to about 11% (*n* = 10) of DKA episodes in our series. High-dose steroids raise blood glucose by inducing insulin resistance and promoting hepatic gluconeogenesis. Cancer patients frequently receive steroids as part of chemotherapy protocols, as antiemetics, or to manage immune-related adverse events. Close monitoring of glucose and proactive counseling of patients with known diabetes on how to adjust their medication therapy in response to high dose steroids may help prevent DKA in this patient population.

The outcomes of DKA in our cancer patients were notably worse than typically seen in non-cancer populations. The in-hospital mortality rate of 16% (*n* = 15) in our study far exceeds the <1% mortality reported in general DKA series and even exceeds the 5–10% mortality observed historically in DKA among the elderly or critically ill [26,27]. Among our patients, death was attributable not to DKA or its usual sequelae, but rather to the severity of concomitant illnesses and advanced malignancies. Most deaths occurred in individuals with widely metastatic cancer who had DKA as one component of multi-organ deterioration. A recent Canadian multicenter study similarly reported a 30% hospital mortality in cancer patients with DKA or HHS, with all deaths occurring in those with advanced cancer [16]. These observations highlight that while aggressive DKA treatment can reverse the metabolic crisis, the underlying oncologic prognosis often drives patient survival.

In addition, we found that the hospital length of stay for DKA in cancer patients was significantly longer than what is typical in general populations. Previous studies in non-cancer cohorts report median hospital stays of about 2–3 days for DKA [28,29]. In contrast, our patients had a median stay of 6 days, and many required more than 1 week hospitalization. This prolonged course may be explained by the need to address underlying cancer issues and other complications and to coordinate specialized care. It suggests that DKA in a cancer patient cannot be viewed as an isolated event, but rather as a syndrome intertwined with their oncologic disease that often mandates multidisciplinary management. The resource implications are significant, and preventive measures to avoid DKA could improve healthcare utilization.

About one-third of our cohort had underlying poorly controlled diabetes at baseline, defined as HbA1c > 9% (75 mmol/mol). However, despite average diabetes duration of >10 years in patients with type 1 and type 2 diabetes, 63% and 18% of patients, respectively, did not proactively adjust insulin therapy and/or notify their healthcare provider with worsening glucose control prior to DKA presentation. This study highlights the importance of taking a proactive approach in cancer patients with known diabetes to provide education on diabetes survival skills including adequate counseling on adjustments that will be needed to their diabetes regimen as they begin cancer treatment.

Interestingly, the use of continuous glucose monitors (CGMs) was low in our patients with pre-existing diabetes. This may be an opportunity for prevention of DKA, as the use of CGMs has been associated with decreased rates of DKA in both pediatric and adult populations [30,31].

Our study has several limitations. It was retrospective and the sample size was small. The sample size, while the largest reported to date for this scenario, is still relatively modest for drawing definitive conclusions. Furthermore, while our univariate survival analysis identified diabetes type a predictor of mortality, the small number of mortality events (*n* = 15) limited our statistical power and precluded a meaningful multivariate analysis to adjust for confounding factors. Also, all the patients were treated at a single large comprehensive cancer center; therefore, the findings may not be generalizable to cancer patients treated in other settings.

Another limitation is that we relied on documentation to identify precipitating factors; in some cases, attribution can be subjective, though we attempted to mitigate this by using objective data such as elevated WBC count/positive culture supporting infection, elevated HbA1C supporting baseline poor diabetic control, and a review of EMR and pharmacy records in tandem with physician notes. Despite these limitations, our study provides important insights into an under-recognized clinical problem and highlights actionable targets for intervention.

We think our results provide important information on the epidemiology of DKA in adults with cancer. In many ways, precipitating factors for DKA differed from what has been described previously for the general population, so strategies for prevention should be tailored to patients being treated for cancer. This study highlights the importance of close monitoring and counseling for patients on cancer therapies that are associated with DKA, to help with earlier identification and intervention. Our results highlight an important need in patients with cancer to help prevent DKA, though proactive counseling, active monitoring of glucose for cancer patients with known diabetes receiving therapies known to cause hyperglycemia, and anticipatory adjustments to insulin therapy for patients placed on high dose steroids. Oncologists and others treating cancer should be made aware of the risk of euglycemic DKA with SGLT2 inhibitors in patients with poor intake or volume depletion. Also, systems should be implemented for initiation of basal insulin therapy when patients with absolute insulin deficiency are admitted to hospital. Addressing these knowledge gaps among providers and timely involvement of endocrine specialty services can help address this important patient safety issue.

## 5. Conclusions

We characterized the precipitating factors and outcomes of DKA in adults with cancer in our hospital, and found they differ significantly from those in the general diabetic population. In this largest to date retrospective cohort, the predominant precipitant of DKA was adverse effects of cancer-related medications, principally immune checkpoint inhibitors, high-dose glucocorticoids, and SGLT2 inhibitors. Other important factors were inadequate insulin therapy and infection. The consequences of DKA in cancer patients were serious, with prolonged hospitalizations and high mortality, especially in those with advanced malignancy. These outcomes highlight that DKA in a cancer patient is a complex syndrome intertwined with their underlying malignancy. Timely recognition, proactive counseling, and a tailored multidisciplinary approach may help improve patient safety and outcomes. Future studies are warranted to elucidate further specific risk factors for DKA in this population and to evaluate targeted interventions for DKA prevention.

## Figures and Tables

**Table 1:** Baseline Demographics and Characteristics.

Characteristic	Total (*n* = 91)	Type 1 Diabetes (*n* = 19, 21%)	Type 2 Diabetes (*n* = 45, 49%)	Drug- Induced Diabetes (*n* = 27, 30%)	*p*-Value
Age, years—Median (IQR)	63 (44–69)	62 (40–67)	63 (49–69)	62 (41.5–70.5)	0.77
Female sex—no. (%)	42 (46%)	9 (47%)	21 (47%)	12 (44%)	1.00
HbA1C checked—no.	74 (81%)	14 (74%)	38 (84%)	22 (81%)	—
Baseline HbA1C %– Median (IQR)	8.2 (7.3–10.4)	8.1 (7.3–9.7)	8.1 (7.1–12)	8.4 (7.3–9.7)	0.82
HbA1C > 9%—no. (%) (*n* = 74)	29 (39%)	5 (36%)	16 (42%)	8 (36%)	0.81
Duration of diabetes, years—Median (IQR)	5 (0–18)	25 (12–35)	11 (1–19)	0 (0–0)	<0.001
Lack of diabetes survival skills—no. (%)	22 (24%)	12 (63%)	8 (18%)	2 (7%)	<0.001
CGM use before presentation	4 (4%)	3 (16%)	1 (2%)	0 (0%)	-

**Table 2:** Characteristics of Malignancy.

Characteristic	Total (*n* = 91)	Type 1 Diabetes (*n* = 19, 21%)	Type 2 Diabetes (*n* = 45, 49%)	Drug-Induced Diabetes (*n* = 27, 30%)	*p*-Value
**Cancer Diagnosis**					0.053
Gastrointestinal Malignancy	18 (20%)	3 (16%)	9 (20%)	6 (22%)	
Dermatological Malignancy	14 (15%)	3 (16%)	4 (9%)	7 (26%)	
Hematological Malignancy	13 (14%)	3 (16%)	9 (20%)	1 (4%)	
Genitourinary Malignancy	10 (11%)	1 (5%)	4 (9%)	5 (19%)	
Breast Cancer	7 (8%)	1 (5%)	5 (11%)	1 (4%)	
Thoracic Malignancy	7 (8%)	0 (0%)	5 (11%)	2 (7%)	
Head & Neck Malignancy	5 (5%)	4 (21%)	0 (0%)	1 (4%)	
Gynecological Malignancy	5 (5%)	0 (0%)	3 (7%)	2 (7%)	
Central Nervous System Malignancy	3 (3%)	1 (5%)	1 (2%)	1 (4%)	
Sarcoma	2 (2%)	1 (5%)	1 (2%)	0 (0%)	
Endocrine Malignancy	1 (1%)	0 (0%)	0 (0%)	1 (4%)	
No Malignancy	6 (7%)	2 (11%)	4 (9%)	0 (0%)	
**Cancer Status**					
No Evidence of Disease (NED)	8 (9%)	5 (26%)	3 (7%)	0 (0%)	0.009
Stage I	2 (2%)	1 (5%)	1 (2%)	0 (0%)	
Stage II	4 (4%)	0 (0%)	4 (9%)	0 (0%)	
Stage III	2 (2%)	0 (0%)	1 (2%)	1 (4%)	
Stage IV or Metastatic	61 (67%)	11 (58%)	25 (56%)	25 (93%)	
Unknown Stage/Staging not available	14 (15%)	2 (11%)	11 (24%)	1 (4%)	

For patients with preexisting type 1 diabetes, 68% of patients (*n* = 13) were using multiple daily injections of insulin, while 26% patients (*n* = 5) were on insulin pump therapy prior to admission and 5% of patients (*n* = 1) were on GLP1 agonist in addition to multiple daily injections. Among patients with type 2 diabetes, 87% (*n* = 39) were being treated with at least one antihyperglycemic medication. This included 38% of patients (*n* = 17) on oral agents and/or GLP-1 agonists without insulin. A total of 13% of patients (*n* = 6) with type 2 diabetes were not on any diabetes medication prior to the DKA (Table 3).

**Table 4:** Drug usage in patients with DKA.

Drug Class	Total (*n* = 91)	Type 1 Diabetes (*n* = 19, 21%)	Type 2 Diabetes (*n* = 45, 49%)	Drug Induced Diabetes (*n* = 27, 30%)	*p*-Value
Immunotherapy use—no. (%)	26 (29%)	0 (0%)	3 (7%)	23 (85%)	<0.001
Steroid use (*n* = 88)—no. (%)	10 (11%)	1 (6%)	7 (16%)	2 (7%)	0.49
SGLT2 Inhibitor use—no. (%)	12 (13%)	0 (0%)	12 (27%)	0 (0%)	<0.001
Antidepressant use—no. (%)	21 (23%)	4 (21%)	9 (20%)	8 (30%)	0.80

**Table 5:** DKA Characteristics.

Characteristic	Total (*n* = 94)	Type 1 Diabetes (*n* = 20)	Type 2 Diabetes (*n* = 46)	Drug- Induced Diabetes (*n* = 28)	*p*-Value
DKA Severity—no. (%)					
Mild	39 (41%)	7 (35%)	23 (50%)	9 (32%)	0.55
Moderate	26 (28%)	7 (35%)	9 (20%)	10 (36%)	
Severe	29 (31%)	6 (30%)	14 (30%)	9 (32%)	
Length of Hospitalization (days)—Median (IQR)	6 (3–13)	4 (3–6)	8 (4–16)	6 (4–10)	0.019

**Table 6:** Mortality outcomes.

Characteristics	Total (*n* = 94)	Type 1 Diabetes (*n* = 20)	Type 2 Diabetes (*n* = 46)	Drug-Induced Diabetes (*n* = 28)	*p*-Value
In-Hospital Death—no. (%)	15 (16%)	0 (0%)	11 (24%)	4 (14%)	0.055
Death within 30 Days After Discharge—no. (%)	5 (5%)	1 (5%)	3 (7%)	1 (4%)	0.861
Death During Study Period—no. (%)	40 (44%)	9 (47%)	21 (46%)	10 (36%)	0.60
Time to Death (Weeks) Among patients who died—Median (IQR)	9.05 (2–24.15)	24.3 (15.1–38.7)	3 (0.5–12.0)	8.5 (2.0–28.9)	0.009

**Table 7:** Univariate survival analysis—in hospital mortality.

Measure	Total Events	*p*-Value	Hazard Ratio (95% CI)
Length of the hospital stay	40:15	0.79	1.00 (0.98–1.02)
Cancer staging		0.39 *	
NED	3:0		
Stage II	1:0		
Stage IV	4:1		
Metastatic	28:13		
Cancer staging			
Non-Metastatic	8:1	0.10	Reference level
Metastatic	28:13		4.4 (0.6–33.9)
Age	40:15	0.36	1.02 (0.98–1.05)
DM type		0.035 *	
T1DM	9:0		Ref
T2DM	21:11	0.99	1.7 × 10^7^ (0–NE)
Drug induced diabetes	10:4	0.99	1.1 × 10^7^ (0–NE)
Classification of Provoking fact			
Drug induced			
No	19:6	0.52 *	
Yes	21:9		
Infection related			
No	27:11	0.48 *	
Yes	13:4		
Inadequate exogenous insulin			
No	31:14	0.08 *	
Yes	9:1		
Other			
No	39:14	0.008 *	
Yes	1:1		

* *p*-value marked with an asterisk is obtained from the log-rank test; all other *p*-values are derived from the Coxph model.

**Table 8:** Univariate survival analysis—30 day mortality after discharge.

Measure	Total Events	Hazard Ratio (95% CI)	*p*-Value
Overall	25:5		
Length of the hospital stay		1.00 (0.98–1.03)	0.90
Cancer staging			
NED	3:0		0.12 *
Stage II	1:0		
Stage IV	4:2		
Metastatic	28:2		
Age	25:5	1.01 (0.95–1.08)	0.75
DM type			
T1DM	9:1	Ref	0.61 *
T2DM	10:3	2.82 (0.29–27.18)	0.81
Drug induced diabetes	6:1	1.50 (0.09–24.06)	0.08
Classification of Provoking fact			
Drug induced			
No	13:3		0.66 *
Yes	12:2		
Infection related			
No	16:2		0.23 *
Yes	9:3		
Inadequate exogenous insulin			
No	17:4		0.55 *
Yes	8:1		
Other			
No	25:5		

* *p*-value marked with an asterisk is obtained from the log-rank test; all other *p*-values are derived from the Coxph model.

**Table 9:** DKA provoking factors.

Provoking Factors	Total (*n* = 94)	Type I Diabetes (*n* = 20)	Type II Diabetes (*n* = 45)	Drug-Induced Diabetes (*n* = 29)
Drug Induced	50 (53%)	1 (5%)	20 (44%)	29 (100%)
Inadequate Exogenous Insulin	34 (36%)	17 (85%)	15 (33%)	2 (7%)
Infection Related	20 (21%)	4 (20%)	14 (31%)	2 (7%)
New DM unrelated to cancer treatment	1 (1%)	0 (0%)	1 (2%)	0 (0%)
Unknown (Misc.)	3 (3%)	0 (0%)	3 (7%)	0 (0%)
Number of Provoking Factors—1	79 (84%)	18 (90%)	37 (82%)	24 (83%)
Number of Provoking Factors—2	14 (15%)	2 (10%)	7 (15%)	5 (17%)
Number of Provoking Factors—3	1 (1%)	0 (0%)	1 (2%)	0 (0%)

## Data Availability

Data are available on reasonable request. All data relevant to the study are included in the article. All data will be available upon request.
